# Leishmaniasis en Bolivia, revisión y estado actual en Tarija, frontera con Argentina

**DOI:** 10.7705/biomedica.4990

**Published:** 2020-08-20

**Authors:** Juan Sergio Mollinedo, Zoraida Mollinedo, Marcelo Magne, Wilson J. Gironda, Óscar D. Salomón

**Affiliations:** 1 Instituto de Salud y Medio Ambiente, Asociación Privada de Laboratorios, La Paz, Bolivia Instituto de Salud y Medio Ambiente Asociación Privada de Laboratorios La Paz Bolivia; 2 Carrera de Medicina, Cátedra de Parasitología, Universidad Amazónica de Pando, Pando, Bolivia Universidad Amazónica de Pando Pando Bolivia; 3 Programa de Leishmaniasis, Secretaría Departamental de Salud Tarija, Ministerio de Salud, Tarija, Bolivia Secretaría Departamental de Salud Tarija Ministerio de Salud Tarija Bolivia; 4 Sociedad Boliviana de Entomología, La Paz, Bolivia Sociedad Boliviana de Entomología La Paz Bolivia; 5 Instituto Nacional Medicina Tropical, Puerto Iguazú, Misiones, Argentina Instituto Nacional Medicina Tropical Puerto IguazúMisiones Argentina

**Keywords:** leishmaniasis, Leishmania braziliensis, Bolivia, Argentina, leishmaniasis, Leishmania braziliensis, Bolivia, Argentina

## Abstract

**Introducción.:**

En 1997, en el departamento de Tarija, Bolivia, situado en la frontera con Argentina, se notificó por primera vez la presencia de pacientes con úlceras en las partes descubiertas de la piel, cuyas características clínicas y epidemiológicas correspondían a leishmaniasis.

**Objetivo.:**

Describir y comprobar la presencia de leishmaniasis en Tarija, sexto departamento endémico en Bolivia.

**Materiales y métodos.:**

Se hizo un estudio del brote (noviembre de 1998 a diciembre de 2002) y un estudio longitudinal (1997 a 2018) en humanos; además, se capturaron Phlebotominae y potenciales reservorios.

**Resultados.:**

Se registraron 1.250 pacientes de leishmaniasis; 190 y 249 casos, en los brotes de 1998 y 2002, respectivamente, con periodos interepidémicos de 37 casos como promedio anual. El 68 % de los enfermos eran pobladores migrantes del altiplano asentados en viviendas precarias cercanas al bosque residual; el sexo predominante fue el masculino (2/1). El grupo etario económicamente activo (15 a 49 años) fue el más afectado (363/584, 62 %). Hubo 124/584 (21 %) menores de 15 años, 33/584 de menos de cuatro años. En 51/584 (8,7 %) pacientes se presentaron lesiones mucosas. Se aisló y caracterizó *Leishmania (V.) braziliensis* de úlceras mucosas de perros enfermos y se capturó abundantemente la especie antropofílica *Nyssomyia neivai*, incriminada como probable vector.

**Conclusiones.:**

En 1997 se comprobó por primera vez la presencia de leishmaniasis tegumentaria en el municipio de Bermejo y, en el 2018, ya se había extendido a cuatro municipios: Padcaya, Caraparí, Entre Ríos y Yacuiba, en dirección noreste del departamento de Tarija.

## Abstract (en)

**Introduction:**

In the department of Tarija in the Bolivian-Argentine border, human cases with ulcers on uncovered parts of the skin plus clinical and epidemiological characteristics related to leishmaniasis were reported for the first time in 1997.

**Objective:**

To describe and to verify the presence of leishmaniasis in Tarija, sixth endemic department in Bolivia.

**Materials and methods:**

We conducted both an outbreak study (November, 1998, to December, 2002) and a longitudinal study (1997 to 2018) in humans, as well as captures of Phlebotominae and potential reservoirs.

**Results:**

A total of 1,250 patients were registered; in the outbreaks, 190 (1998) to 249 cases (2002) were reported and inter-epidemic periods with 37 cases as an annual average; 68% of the patients were highland migrants who inhabited precarious housing near residual forests. The predominant sex was male (ratio 2:1); the most affected group (363/584 cases, 62%) was the economically active (15 to 49 years old); 124/584 cases (21%) were children under 15 years old, 33/584 of them were under 4 years old; 51 patients/584 (8.7%) had mucosal lesions. *Leishmania (V.) braziliensis* was isolated and characterized from mucous ulcers of sick dogs. *Nyssomyia neivai,* an abundant anthropophilic species incriminated as a probable vector, was captured.

**Conclusions:**

The initial 1997 leishmaniosis presence in the municipality of Bermejo had spread out over four municipalities in 2018 (Padcaya, Caraparí, Entre Ríos, and Yacuiba), northeast of the department of Tarija.

Las leishmaniasis tienen distribución mundial en 102 países, con una incidencia anual estimada de 0,7 a 1 millón de casos de la leishmaniasis tegumentaria, y de 200.000 a 400.000 casos de la visceral ^1^.

Son infecciones parasitarias con significativa diversidad clínica y epidemiológica, de amplia distribución geográfica en el Viejo y el Nuevo Mundo, transmitidas por varias especies de vectores flebotomíneos (Psychodidae, Phlebotominae) y asociadas con diferentes parásitos del género *Leishmania* y huéspedes reservorios que propician ciclos de transmisión distintos ^2^. Las diversas especies de *Leishmania* morfológicamente semejantes presentan grados variables de especificidad en cuanto a sus huéspedes invertebrados ^3^. El desarrollo de la enfermedad depende de factores inherentes a la reacción inmunitaria del huésped humano, al agente etiológico y al vector responsable de la transmisión.

Las zonas tropicales y subtropicales del Estado Plurinacional de Bolivia son áreas endémicas con presencia de leishmaniasis cutánea y mucocutánea, así como de casos esporádicos de leishmaniasis visceral ^4^^,^^5^.

Las lesiones atribuidas a las leishmaniasis cutánea y mucosa se conocen desde la época precolonial. En 1906, Pardo-Valle determinó como áreas endémicas las márgenes de los ríos de cinco departamentos (La Paz, Beni, Pando, Santa Cruz y Cochabamba) ^6^ y diversos estudios confirman que las áreas endémicas se extienden desde las laderas subandinas altas (Yungas) y bajas (300 a 2.000 m), asociadas con los tributarios de la cuenca amazónica, hasta las tierras bajas tropicales de la Amazonia (200 m) ^5^^,^^6^.

En la zona fronteriza entre Bolivia y Argentina, en la ecorregión compartida por ambos países de bosque tropical y subtropical húmedo y vegetación xerófila (chaco), se han registrado casos de leishmaniasis tegumentaria desde 1916 hasta el primer brote epidémico en la década de 1980 ^7^^-^^9^ y siete casos esporádicos de leishmaniasis visceral entre 1923 y 1932, incluidos dos migrantes de zonas endémicas para esta leishmaniasis en Europa, sin nuevos registros hasta 2008 ^10^^,^^11^.

Otra referencia inespecífica señala que, en la zona de los ríos Bermejo y Grande de Tarija, los trabajadores de empresas de prospección de petróleo (Richmond Levering, 1918, y Standard Oil, 1922), se enfermaban con “úlceras redondeadas, bordes elevados, en partes descubiertas de la piel” (Harvey B. Technical report of hygiene digest on work in Bermejo river Tarija - Bolivia. Standard Oil. 1922; sin publicarse).

## Materiales y métodos

### Zona de estudio

El municipio de Bermejo alberga la ciudad con el mismo nombre, la más austral de Bolivia (22º 35’ 24” S y 64º 14’ 55” O); su origen está ligado a las actividades petroleras (1918) y a la industria de la caña de azúcar (1972).

El territorio del municipio se encuentra en una región mesopotámica entre los ríos Bermejo y Tarija, y conforma el llamado Triángulo del Sur, con una altura media de 419 m, una temperatura promedio de 22,8 ºC y una precipitación anual promedio de 1.200 mm. La topografía es plana con ligeras ondulaciones y su suelo es de origen aluvial en los ríos que la rodean.

Presenta dos zonas bioclimáticas: una de bosque húmedo templado (bosque tucumano-boliviano) y otra de bosque seco templado en transición a bosque húmedo (bosque chaco-salteño). La primera es más apta para la agricultura y la ganadería, y sus principales cultivos son la caña de azúcar, el maíz y los cítricos. Contaba con una población estimada de 27.372 habitantes en 1998 y de 39.280 en el 2018; el 51 % de los habitantes son hombres, el 78,1 % de la población es urbana y el resto vive en poblaciones periféricas cerca del bosque o de ríos.

**Figura 1:**
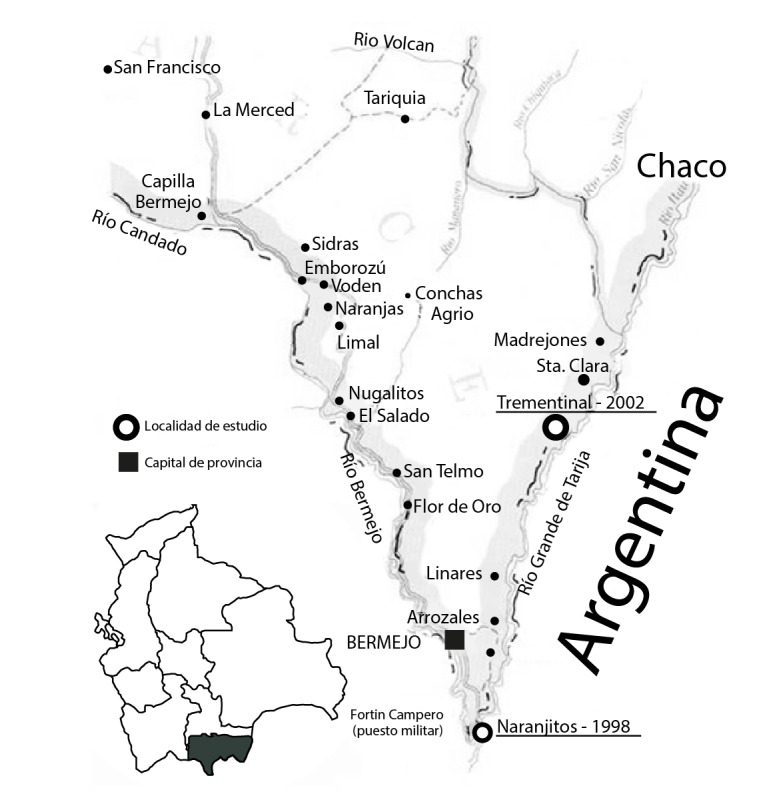
Zona geográfica de estudio de los brotes de leishmaniasis de 1998 a 2002, municipios de Bermejo y Padcaya del Departamento de Tarija. Las localidades estudiadas durante varios años aparecen subrayadas.

La ciudad de Bermejo se encuentra a 80 km de la frontera con Argentina ^12^ (figura 1). Cada año hay un importante flujo de migrantes andinos para la zafra de azúcar, que se asientan en viviendas precarias hechas de madera y caña, y con peridomicilio próximo al borde de vegetación densa, las cuales se encuentran dispersas a la sombra del bosque residual o dentro de este, o al borde de los campos de azúcar y a poca distancia de ríos y arroyos.

Los conocimientos y las prácticas de esta población son inadecuadas para la zona en cuanto al manejo del peridomicilio y de la basura orgánica. Tienen hábitos que aumentan la exposición a vectores, como usar poca vestimenta y dormir fuera de sus viviendas; las letrinas están rodeadas de vegetación baja, recogen agua de pozos, arroyos y ríos, y leña para cocinar de galerías de bosque; crían animales dentro y fuera del domicilio (aves, cerdos, perros, gatos, conejos, cabras) y no realizan ningún control de animales sinantrópicos, pues se ha evidenciado la presencia de roedores y marsupiales (Mollinedo S., datos sin publicar) (cuadro 1).

**Cuadro 1:** Número de casos de leishmaniasis cutánea y mucosa por años y edad.

Año	Tipo de lesión	Grupos de edad (años)				
		1 a 4	5 a 14	15 a 49	50 o más	Total
1997	Cutánea	5	4	17	4	30
	Mucosa	0	1	1	0	2
1998	Cutánea	7	32	114	26	179
	Mucosa	0	1	6	4	11
1999	Cutánea	4	7	30	8	49
	Mucosa	0	0	4	2	6
2000	Cutánea	2	1	10	5	18
	Mucosa	0	0	5	3	8
2001	Cutánea	0	2	14	6	22
	Mucosa	0	1	7	2	10
2002	Cutánea	15	40	144	36	235
	Mucosa	0	2	11	1	14
Subtotal leishmaniasis cutánea		33	86	329	85	533
Subtotal leishmaniasis mucosa		0	5	34	12	51
Total		33	91	363	97	584

Fuente: Mollinedo S. Informe anual, Laboratorio Nacional de Parasitología, 2002

### Estudio de foco

A partir de 1997 comenzaron a detectarse casos en el municipio de Bermejo. Después de una lenta y tardía notificación del brote que duró 46 meses, de noviembre de 1998 a diciembre de 2002, su estudio estuvo a cargo del equipo multidisciplinario del Instituto Nacional de Laboratorios de Salud - *Institut de Recherche pour le Developement* (INLASA/IRD), que luego continuó con un estudio longitudinal hasta el año 2018 a cargo del personal local del Servicio Departamental de Salud de Tarija.

### Epidemiología

La historia clínica *ad hoc* para el estudio del brote se basó en un examen físico de los enfermos que acudieron espontáneamente a los servicios del sistema de salud y en la detección activa en las zonas de recolección de caña y los domicilios rurales; asimismo, se comenzaron a dar capacitaciones anuales previa validación de cuatro manuales ^13^^-^^16^, seguidas de la supervisión de todo el personal de los centros de salud de la zona.

*Detección de pacientes*. Se consideraba como caso clínico sospechoso al paciente con uno de estos signos:

a) úlcera no provocada por traumatismo;

b) úlcera con más de tres semanas de evolución;

c) úlcera característica (forma redondeada, bordes levantados, rojiza, fondo limpio, poco dolorosa);

d) lesiones satélites;

e) linfadenopatía regional, y

f) lesiones vegetantes o nodulares (13-16).

*Toma de muestra.* Se empleó la técnica de raspado de la lesión con un escarbadientes previamente esterilizado, después se limpiaba y se pinchaba el borde activo de la lesión mediante rotación del palillo. El material obtenido se extendía en un portaobjetos para posteriormente fijarlo (metanol), teñirlo (panóptico rápido) y observarlo bajo el microscopio. La toma de la muestra de los tres lugares más representativos de cada úlcera se repitió. Este procedimiento es el adoptado por los centros de salud de primer nivel (baja complejidad) ^4^^,^^15^^,^^16^.

Se utilizó también la punción y la aspiración mediante el uso de jeringas cargadas con solución salina tampón con fosfato (PBS) en tres regiones del borde de la lesión para cultivar en el medio de cultivo bifásico NNN (Novy, McNeal, Nicolle) ^13^^-^^16^.

*Métodos de diagnóstico.* Se emplearon los frotis, los cuales se observaban en los portaobjetos y con objetivos embebidos en aceite de inmersión bajo el microscopio. Asimismo, se recurrió al cultivo; los medios sembrados se trasladaban al Laboratorio Nacional de Parasitología del Instituto Nacional de Laboratorios de Salud, manteniéndolos a 27 ºC y se observaba su crecimiento cada 48 horas durante un periodo de 20 días. Se empleó también la inoculación en hámsteres que, una vez inoculados con muestras procesadas de pacientes, eran transportados al laboratorio nacional para su observación por un periodo de seis meses ^13^^-^^16^.

*Tratamiento*. Los pacientes con diagnóstico clínico o parasitológico positivo fueron remitidos al sistema local de salud para su tratamiento con antimoniales pentavalentes por vía intramuscular ^14^.

### Muestreo de Phlebotominae

En el periodo de transición a la época de lluvias (octubre de 1998), se hicieron capturas de insectos mediante dos métodos: con cebo humano protegido y con trampas CDC de luz.

Se establecieron cuatro áreas diferentes de captura:

1) bosques xéricos (durante una noche, tres transectos con diez trampas de luz cada uno);

2) campo de caña (durante una noche, un transecto con diez trampas de luz);

3) hábitat rural cubierto de vegetación (durante una noche, un transecto con seis trampas de luz), y

4) establos de cerdos (durante una noche, una trampa de luz por establo).

Los flebotomíneos fueron procesados e identificados *in situ* y, posteriormente, transportados al Instituto Nacional de Laboratorios de Salud ^17^^,^^18^. Una segunda captura con cebo humano protegido y trampas Shannon se hizo en Naranjitos, Alto Calama y San Telmo (julio de 2003).

### Reservorios potenciales

Se investigaron 348 perros (8,6 % de áreas rurales y 91,4 % del área urbana) ^19^ mediante frotis de lesiones sospechosas en hocicos, orejas y genitales. Se capturaron mamíferos silvestres con trampas Sherman colocadas al atardecer en transectos de 5, 10, 15 y 20 estaciones con separaciones de 2 m, en ocho hábitats diferentes (plantación de bananos, campo, plantación de maíz, bosque chaqueño, plantación de caña de azúcar de un metro de altura, pastizales, al aire libre y en el interior de las casas).

### Aislamiento y tipificación

Uno de los cuatro perros con úlceras en hocico, orejas y genitales encontrados en la zona, fue transportado al Laboratorio Nacional de Parasitología para el aislamiento y caracterización de los parásitos.

Los parásitos del cultivo de la úlcera del hocico del perro se caracterizaron mediante ocho sistemas isoenzimáticos y se compararon con seis cepas de referencia: glucosa fosfatoisomerasa (GPI, EC 5.3.1.9), glucosa-6-fosfato deshidrogenasa (G6PD, 1.1.1.49), isocitrato deshidrogenasa (IDH, EC 1.1.1.42), 6-fosfo-gluconato deshidrogenasa (6PGD, EC 1.1.1.44), glutamato oxaloacetato transaminasa (GOT, EC 2.6.1.1.), malato deshidrogenasa NAD (MDH, EC 1.1.1.37), nucleósido hidrolasa, sustrato inosina (NHi, EC 3.2.2.) y diaforasa (DIA, EC 1.6.99.2).

El método para la extracción de ADN fue el propuesto por Breniere, *et al*. ^20^^,^^21^ La extracción de ADN se llevó a cabo en la sangre del perro (conservada en guanidina) y el sedimento parasitario obtenido del cultivo. Estas muestras de ADN se hibridaron posteriormente con sondas de *L. (V) braziliensis* y *Leishmania (L.) amazonensis*. La detección por PCR de *Leishmania* se realizó en un cultivo de parásitos tomados de una muestra del perro, según Breniere, *et al.*, ^21^ y con los cebadores L1-5’CCT ACC CAG AGG CCT GTC GGG-3’ y L2-5’TAA TAT AGT GGG CCG CGC AC-3’ adquiridos en el Laboratorio Eurogentec (Scraing, Belgium).

Los productos de PCR se analizaron por electroforesis en geles de agarosa al 1,5 % en TAE X 0,5 y se visualizaron en tinción con bromuro de etidio. Cada protocolo incluyó controles negativos y positivos, y se utilizó como plantilla agua libre de ADN y ADN de parásito purificado, respectivamente.

*Etiquetado e hibridación.* Se utilizaron tres sondas sondas ADNk (ADN del cinetoplasto) específicas para *Leishmania*: *L. (L.) mexicana* (MNYC/BZ/ M379), *L. (L) chagasi* (MHOM/BR/74/PP75) y *L. (V) braziliensis* (MHOM/ BO/90/CG), producidas mediante amplificación por PCR ^21^ utilizando los cebadores L1 y L2 adquiridos en el Laboratorio Eurogentec (Seraing- Bélgica). Los productos de la PCR se transfirieron a una membrana de nailon; la marcación de las sondas y la hibridación se hicieron con el sistema de detección del gen de quimioluminiscencia mejorado (ECL Amersham, Buckinghamshire, Reino Unido) según las instrucciones del fabricante.

### Estudio serológico y PCR

En julio de 2003 se encuestaron 801 personas y se tomaron 732 muestras sanguíneas en ocho comunidades estables de la zona (Cajones, San Antonio, Santa Clara, Trementinal, San Telmo, Quebrada Chica, Alto Calama y Naranjitos). para la detección de anticuerpos contra *Trypanosoma cruzi* (ELISA- Tc y IFI-Tc), contra *Leishmania* spp*.* (ELISA-SLA y IFI-L) y contra *Leishmania infantum chagasi* (ELISA rk-39) ^22^.

### Consideraciones éticas

Todas las autoridades de las distintas comunidades firmaron un consentimiento informado para realizar los estudios clínicos, recolectar una muestra de sangre, tomar biopsias y publicar los resultados de forma anónima. Todas las actividades están inmersas dentro de la normativa del Programa Nacional de Leishmaniasis, aprobadas por el Ministerio de Salud.

## Resultados

### Estudio del brote (1997 a 2002)

*Aspectos clínicos y de diagnóstico.* Inicialmente, se revisaron las fichas clínicas y se hizo la búsqueda activa de los casos registrados con anterioridad (32 notificados en 1997 y 190 en 1998) en las poblaciones de Naranjitos, Campo Grande, Porcelana, Colonia Linares, Barredero, Trementinal, San Telmo, El Nueve y la Ciudad de Bermejo. Se hizo un nuevo examen físico general, y uno de piel y de las mucosas oral y nasal, a 193/221 (87 %) de los pacientes inicialmente registrados (se tomó frotis de ocho pacientes con úlceras activas sin tratamiento y sin contaminación microbiana, y se hallaron formas amastigotas de *Leishmania* spp*.* en cuatro de ellos).

Debido a la capacitación e implementación de los manuales, se comenzaron a optimizar los tiempos de diagnóstico y tratamiento ^23^.

En este periodo de estudio, se presentaron dos brotes epidémicos en 1998 (190 casos, 8,19 % del total nacional) y en 2002 (249 casos, 9,8 % del total nacional). En total, se registraron 584 enfermos: 533 (91,26 %) con leishmaniasis cutánea y 51 (8,73 %) con mucocutánea. El sitio probable de infección fue la zona rural del municipio de Bermejo para todos los pacientes, asimismo, todos trabajaban o vivían en la zona de cultivo de la caña de azúcar y en el bosque.

La mayoría de los enfermos eran hombres, 388/584 (66,4 %), y 196/584 (33,6 %) eran mujeres, con una proporción de 2:1. Se observó que 363/584 (62 %) pacientes tenían edades entre los 15 y los 49 años; 97/584 (16,6 %) eran mayores de 50 años, 33/584 (5,6 %), menores de 4 años, y 91/584 (15,5 %) tenían entre 5 y 15 años.

El examen clínico reveló lesiones simples en diferentes estados evolutivos (úlceras activas o cicatrices), con diámetro promedio de 2,0 cm (1 a 8 cm).

De los 533 pacientes con leishmaniasis cutánea, 485 tenían una única lesión (239 en miembros inferiores, 129 en miembros superiores, 68 en tronco y abdomen, y 49 en cara); los restantes 55 pacientes tenían úlceras en múltiples localizaciones.

### Aspectos epidemiológicos

El 68 % de 584 de los enfermos eran migrantes temporales para la zafra de caña de azúcar procedentes de la zona andina (Potosí y otras regiones donde no existe leishmaniasis) y el 32 % de 584 restante era nativo de la región. El brote de 1998 se desarrolló en la localidad de Naranjitos (municipio de Bermejo, 15 km al sur de la ciudad de Bermejo) y, el del 2002, en Trementinal (municipio de Padcaya, 50 km al norte de la ciudad de Bermejo). La distribución mensual de la detección de casos evidenció dos picos de incidencia, el primero en julio (comienzo de la cosecha de caña de azúcar) y el segundo en septiembre (final de la cosecha).

### Muestreo de Phlebotominae

Se capturaron 189 especímenes (109 hembras, 80 machos) pertenecientes a tres especies: *Nyssomyyia neivai* (Pinto 1926) ^17^^,^^18^; *Migonemyia migonei* (França), ambas especies antropofílicas, y *Evandromyia cortelezzii* (Brethes); el 92,6 % de las hembras fueron *Ny. neivai.* Estas especies habitan en los parches residuales de bosque que rodean los cultivos y viviendas, en bosques de galería de los ríos principales y sus afluentes, y en áreas de cultivo y periferia de las viviendas ^17^. Localmente, estos insectos se conocen como ‘plumillas’ (pequeñas plumas).

### Potenciales reservorios

A lo largo de 9.716 horas de colocación de trampas, se capturaron 148 especímenes de 9 especies de mamíferos ^19^ y, mediante claves dicotómicas ^24^, se identificaron como dos especies de roedores introducidas (*Mus musculus* y *Rattus rattus*) y siete especies nativas (*Calomys fecundus*, *Akodon* sp., *Balomys* sp., *Oligoryzomys destructor*, *Oxymicterus* sp., *Oxymycterus paramensis* y *Thylamys venustus*). En los mamíferos silvestres capturados no se pudieron hacer otros exámenes debido a la cuarentena por un brote de hantavirus.

En el meticuloso examen clínico-veterinario de los 32 perros, siete tenían lesiones sospechosas en orejas, hocicos y extremidades; en los frotis de cuatro de ellos se detectaron formas amastigotas de *Leishmania* sp p., y uno de los perros se trasladó al Laboratorio Nacional de Parasitología para asegurar el aislamiento y la tipificación del parásito ^19^.

### Aislamiento y caracterización

**Figura 2:**
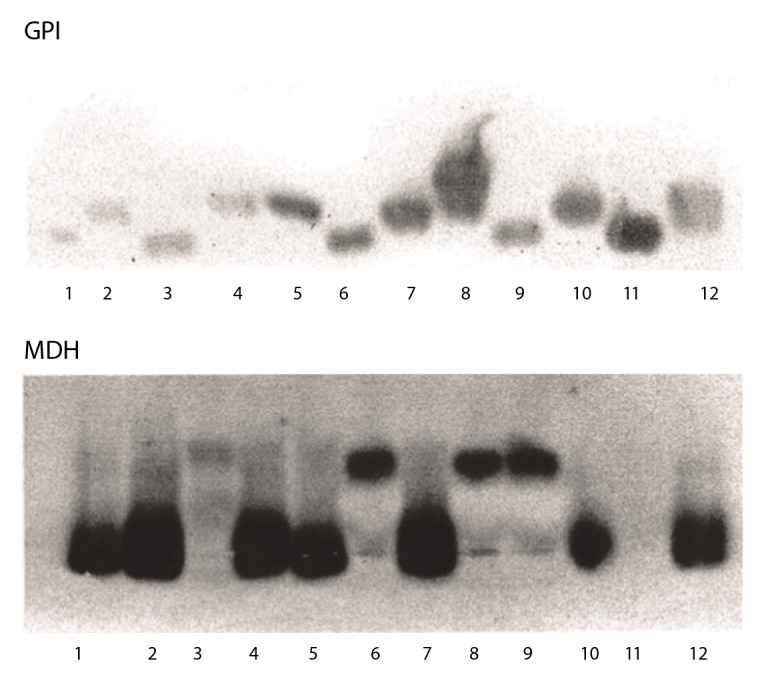
1: LPZ; 2: cepa del perro; 3: leishmaniasis visceral 135 (complejo *L*. mexicana); 4: cepa del perro; 5: M2903 (complejo *L*. *braziliensis*); 6: *L*. *infantum*; 7: cepa del perro; 8: cepa del paciente a; 9: PP75 (complejo *L*. *donovani*); 10: cepa del perro; 11: PH8 (complejo *L*. *mexicana*); 12: cepa del paciente b.

La caracterización con ocho sistemas enzimáticos y seis cepas de referencia, permitió observar que, con los sistemas GPI y MDH, la cepa de perro (carriles 2; 6; 9; 12) tenía una migración similar a la cepa de referencia M-2903 (complejo *L. braziliensis*) (carril 5) (figuras 2 y 3).

Fuente: Vargas F, Bastrenta B, Mita N, Flores M. 2003. Informe interno, sin publicarse.

**Figura 3:**
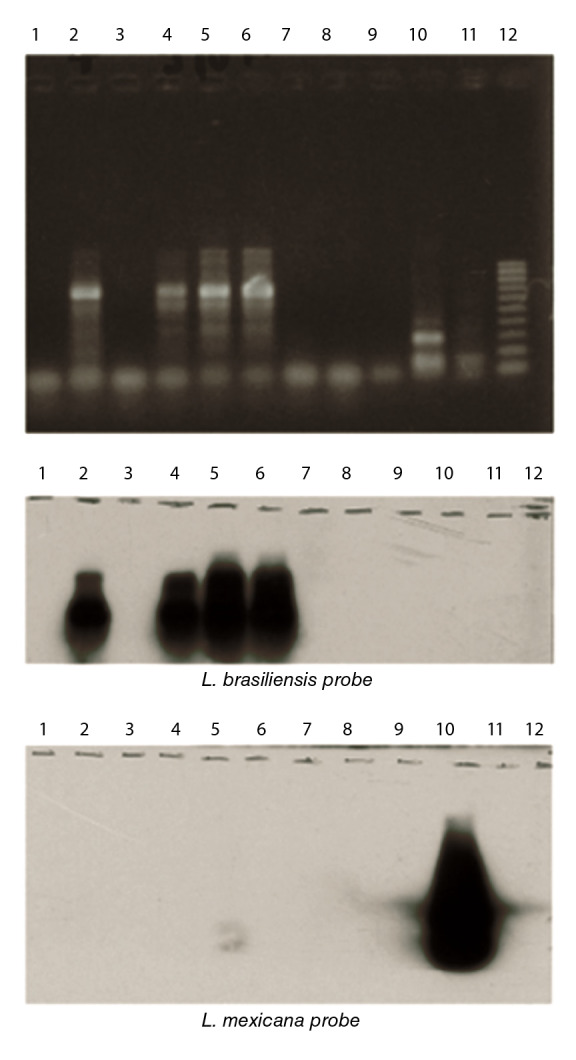
1: control negativo; 2: cepa de referencia (*L.* (*V*) *braziliensis*); 3: control negativo; 4: cultivo de la lesión del perro; 5: cultivo de la lesión del perro; 6: cultivo de la lesión del perro; 7: control negativo; 8: control negativo; 9: control negativo; 10: cepa de referencia (*L*. *mexicana*); 11: cepa de referencia (*L*. (*L*) *infantum*); 12: en la PCR, marcador de bajo peso molecular con baja potencia de 100 pb.

Fuente: Vargas F, Bastrenta B, Mita N, Flores M. 2003. Informe interno, sin publicarse.

### Estudio longitudinal

**Figura 4:**
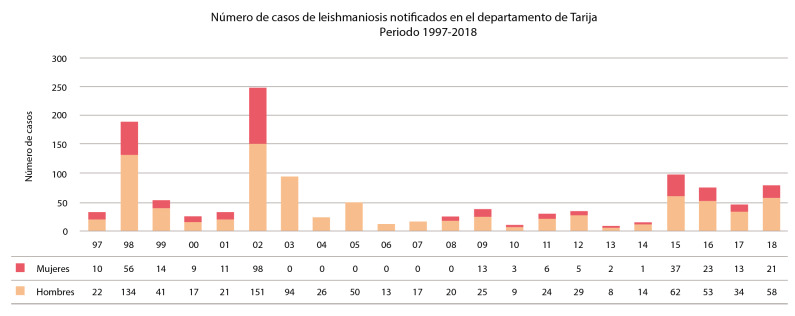
Número de casos de leishmaniasis tegumentaria en el departamento de Tarija, periodo de 1997 a 2018, por años y sexo.

*Aspectos epidemiológicos.* En 22 años de estudio (1997 a 2018), se registraron 1.250 casos; fueron 1.104 (88,3 %) de leishmaniasis cutánea y 146 (11,7 %) de mucocutánea, con un promedio anual interepidémico de 37 casos por año, aproximadamente el 1,6 % del total nacional. El número total de hombres alcanzó los 922 (73,7 %) y se registraron 328 (26,3 %) en mujeres (figura 4) (el valor total de los años 2003 al 2007 no se discriminó por sexo). El porcentaje de casos importados del interior (La Paz, Santa Cruz, Cochabamba y Pando), o del exterior (Argentina), varió mucho según el año (3 a 47 %) y correspondió, en promedio, a 18 % de los casos registrados.

**Figura 5:**
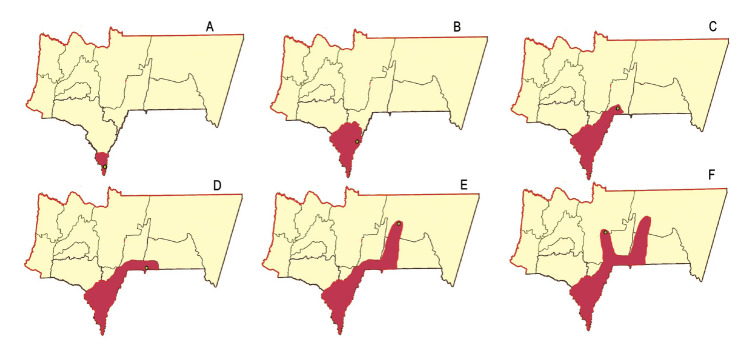
Progresión de casos de leishmaniasis tegumentaria por municipios y años en el departamento de Tarija. A: Naranjitos, primer brote en el año 1998, Municipio de Bermejo. B: Trementinal, segundo brote en el 2002, Municipio de Padcaya. C: Sidras (19,2 % de casos notificados en el 2008), municipio de Caraparí. D: Lapachal (10 % de los casos notificados en el 2011), municipio de Yacuiba. E: El Angosto (6,6 % de los casos notificados en el 2014), municipio de Villamontes. F: Ñaurenda (1,3 % de los casos notificados en el 2016), municipio de Entre Ríos

La notificación de pacientes fue mayor en la época seca (julio a noviembre), con una evolución de dos a cuatro meses de las lesiones ulcerosas, lo que hizo presumir que las infecciones debieron adquirirse principalmente entre abril y julio. Aparentemente, existirían dos modalidades de transmisión de la enfermedad:

- transmisión forestal, que se explicaría por la prevalencia de lesiones únicas adquiridas en el entorno laboral por hombres en edades económicamente productivas, con acentuada prevalencia de lesiones en los miembros inferiores seguidas de las de miembros superiores;
- transmisión nocturna en el peridomicilio y en los bosques próximos a las viviendas, que se evidenciaría por la presencia de lesiones en 33 menores de cuatro años y un número importante de pacientes con lesiones en cara (15 % de los casos).
- Los municipios de Bermejo y Padcaya notificaron el mayor número de casos todos los años, pero a partir del 2008, otros municipios comenzaron a notificar casos autóctonos (figura 5).

### Muestreos de Phlebotominae

Al muestreo inicial de 1997 se añadieron otras dos capturas: la segunda captura (INLASA/IRD; 2003) ^22^, de 2.503 flebotomíneos (2.392 hembras y 111 machos), con predominio de *Ny. neivai*, en Naranjitos, Alto Calama y San Telmo, y la tercera, realizada por el técnico Julio Vidaurre (2015) ^25^ en el barrio San José (periferia de la ciudad de Bermejo) y en la cual se capturaron 22 especímenes: 12 en el domicilio y 10 en el peridomicilio; 20 de ellos eran *Ny. neivai* (13 hembras) y dos eran *Mg. migonei* (1 hembra). En ninguno de los tres estudios realizados se aislaron parásitos de *Leishmania* spp. (cuadro 2).

**Cuadro 2:** Número y porcentaje de *Phlebotominae* muestreados por año en el departamento de Tarija por especie y lugar de captura

Lugar	Año	Especies de Phlebotominae (hembras)					
		*Nyssomyia* *neivai*	*Migonemyia* *migonei*	*Evandromyia* *cortelezzii*	*Lutzomyia* *longipalpis*	Sin clasificar	N° total capturas
Fortín Campero ^16^	1997	101	4 p cf	4 p cf	0	0	109 (80 M)
Calama y San Pedro ^21^	2003	2.302 (92 %)	70 (2,8 %)	9 (0,36 %)	0	122 (4,84 %)	2.381 (122 M)
Bermejo, ciudad ^22^	2015	20 (91 %)	2 (9 %)	0	0	0	22

M: número de machos; p cf: por confirmar

### Estudio serológico y PCR

En la encuesta del 2003, se encontraron 35 casos con un cuadro clínico indicativo de leishmaniasis cutánea, de los cuales se confirmaron siete casos por microscopía y PCR. Se encontró una prevalencia elevada de personas serorreactivas para la enfermedad de Chagas (33,7 % en la prueba de confirmación IFI-Tc). La prevalencia de serorreacción para las leishmaniasis tegumentarias fue de 4,8 % (IFI-L). Se observó un gran número de reacciones cruzadas en la prueba ELISA debido a la coexistencia de leishmaniasis y enfermedad de Chagas en la zona.

Se encontraron cinco (0,7 %) casos probables de leishmaniasis visceral con la prueba ELISA-rk39, en los cuales fue necesaria la confirmación con examen clínico y una nueva toma de muestra. La serología para determinar anticuerpos contra *Leishmania* spp. y la PCR (9,2 % de personas positivas), sugieren la presencia de una población asintomática al inicio del periodo de la zafra ^22^.

## Discusión

El departamento de Tarija no era considerado por el Sistema Nacional de Salud como un área de transmisión documentada de leishmaniasis hasta 1998. El municipio de Bermejo es una nueva área endemo-epidémica de leishmaniasis tegumentaria, que se extendió desde este municipio (1997-1998) hasta los de Padcaya en el 2002, y los de Caraparí, Entre Ríos y Yacuiba en el 2018.

Dicha zona tiene factores humanos, antrópicos y entomológicos, que favorecen el desarrollo de esta endemia. Un factor humano es que los considerables grupos de inmigrantes andinos inmunológicamente vírgenes, sin exposición previa a los efectos potencialmente protectores de la saliva de vectores, constituyen un sustrato fértil para una rápida primoinfección ^26^^,^^27^. Entre los antrópicos, están las condiciones laborales y las viviendas precarias circundadas de bosque residual, la ausencia de servicios básicos, la crianza de animales dentro y fuera del domicilio, y los pozos, ríos y afluentes que atraen a los mamíferos silvestres sinantrópicos. Los factores entomológicos son el predominio de una especie antropofílica, *Ny. neivai*, que ingresa a los domicilios atraída por las lámparas de aceite, la presencia de animales como cerdos y pollos en el peridomicilio, y la posibilidad de que *Mg. migonei* y *Ev. cortelezzii* puedan tener un papel en la transmisión.

*Reservorios potenciales.* Se verificó la presencia de nueve especies de mamíferos (roedores, marsupiales) y de perros con registros de infecciones naturales en diferentes países de América. En el área fronteriza con Argentina, el parásito aislado en la mayor parte de los casos humanos y caninos, y en situaciones epidémicas, también resultó ser *L.* (*V.*) *braziliensis*^28^^,^^29^.

En el primer brote en Naranjitos y aledaños (1998), se registraron 190 casos (prevalencia de 69 por 10.000 habitantes) y, en Trementinal y aledaños (2002), 292 casos (prevalencia de 71 por 10.000 habitantes), con un promedio anual de 37 casos por año en el período interepidémico. En Salta, Argentina, los primeros casos autóctonos de leishmaniasis tegumentaria datan de 1916. En los departamentos de Orán y San Martín, en la ecorregión tropical y subtropical húmeda compartida por los dos países, en localidades próximas a la frontera, se registraron brotes epidémicos entre 1984 y 1987, en 1993, en 1997-1998 y en 2002 ^30^^,^^31^.

Las características clínico-epidemiológicas en Argentina son similares a las del área endémica de Bolivia: grupo etario de enfermos y de diagnóstico de las lesiones cutáneas y mucocutáneas ^31^, y el similar parásito y la misma fauna de vectores antropófilicos*.* La prevalencia de infección resultó más similar entre sexos que la expresión clínica ^31^ y la infección asintomática, que puede llegar el 50,8 % de la población en comunidades con una larga permanencia en zonas hiperendémicas.

En Argentina, así como en este estudio, se observó que el riesgo se asocia también con el trabajo rural o con la vegetación primaria ^32^, el manejo de ganado, la caza, el dormir en espacios abiertos o permanecer en el exterior de la vivienda por más de diez horas, el bañarse en el río, así como la presencia de animales domésticos en el interior del domicilio, las vivienda sin cerramiento ^32^^-^^34^, y la transmisión peridomiciliaria en viviendas hasta los 100 a 200 m de la vegetación primaria o secundaria, incluso en áreas urbanas ^35^^,^^36^ por el efecto de borde ^37^.

Al analizar el riesgo según la actividad de exposición, debe tenerse en cuenta que los vectores tienen diferentes patrones horarios según la estación del año ^38^^,^^39^. Por ello, se determinaron los siguientes tipos de transmisión según el ciclo ^30^:

a) ciclo silvestre, transmisión silvestre;

b) ciclo silvestre, transmisión doméstica por contigüidad o modificación ambiental, y

c) ciclo peridoméstico, transmisión peridoméstica por colonización transitoria de vectores, como se discutirá más adelante.

Presumiblemente, la detección tardía de casos (julio y septiembre) se explicaría por dos razones: el desconocimiento general de la enfermedad en el área y un proceso lento de su confirmación por parte de los servicios de salud locales. Esta situación es similar a la observada en las localidades de Villa Tunari e Ivirgarzama (Chapare, departamento de Cochabamba) en 1986, donde clínica y epidemiológicamente las lesiones evidentes de leishmaniasis cutánea se reportaron durante meses como ‘curación de herida,’ y los médicos recién egresados no conocían la existencia de la leishmaniasis en esta zona (Mollinedo S., datos sin publicar).

En cuanto a los meses de diagnóstico de casos, sin embargo, el patrón anual fue semejante al encontrado en Argentina, lo que se asocia con la dinámica anual de población de vectores y el ciclo intrínseco de incubación en los huéspedes humanos ^40^^,^^41^. Así, los períodos epidémicos en la región fronteriza con Bolivia entre 1986 y 1987, 1990 y 1991, 1996 y 1998, y 2000 y 2004, se presentaron en otoño y primavera y algunos se asociaron con niveles extraordinarios de precipitación y de deforestación ^42^.

Por otra parte, los antecedentes de la fecha probable de infección, evolución de las lesiones y signos clínicos de los 51 pacientes con lesiones mucosas reportados en el estudio de brote, revelaron que las lesiones cutáneas no diagnosticadas ni tratadas por el sistema de salud habían evolucionado durante años, lo que sugiere la existencia de casos de leishmaniasis tegumentaria años antes de la primera notificación. El porcentaje total de casos de leishmaniasis mucosa fue de 11 %.

En Bolivia, la especie más frecuentemente aislada es *L.* (*Viannia*) *braziliensis.* A la primera cepa aislada en Tarija, en un perro en el 2002, se añadieron tres otras cepas aisladas con los genotipos AB1 y AB2 ^35^. En el norte argentino se han confirmado casos poco frecuentes atribuidos a *L.* (*V.*) *guyanensis, L.* (*V.*) *panamensis*^29^ y *L.* (*L.*) *amazonensis*^43^.

La distribución relativa de flebótomos por regiones y su significativa biodiversidad en el Neotrópico, generan diferentes características epidemiológicas. Además, la densidad relativa de flebótomos depende de diferentes factores. Contrasta con las escasas investigaciones realizadas en Tarija (3.203 Phlebotominae de las cuatro especies notificadas), la amplia documentación en la región de Salta (Argentina), fundamentalmente en torno a la presencia de *Ny. Neivai* y *Mg. migonei*, que han sido incriminadas como vectores de *L.* (*V.*) *braziliensis* y se encontraron naturalmente infectadas junto con *Ev. cortelezzii*, *Psathromyia bigeniculata*, *Psa. punctigeniculata* y *Micropygomyia quinquefer*^36^. *Nyssomyia neivai* es la más abundante en bosques secundarios y ambientes peridomésticos asociados con casos humanos; los ciclos estimados de correlación de abundancia entre los ambientes silvestres y peridomésticos permiten inferir que los primeros funcionan como poblaciones fuente de los segundos y que sus poblaciones pueden extinguirse en momentos climáticos no favorables pero persisten durante los brotes. Se encontró, asimismo, una asociación entre la abundancia de *Ny. neivai* y la precipitación del año anterior ^41^^,^^42^. Los mapas predictivos de la distribución de ambos vectores en la región se relacionaron, a su vez, con la precipitación durante el trimestre más cálido y la temperatura media durante el más frío ^41^^-^^43^.

La abundante captura de *Ny. neivai*, especie antropofílica, su infección natural y la concordancia espacio-temporal con la transmisión y los brotes, han permitido incriminarla como vector principal de *L. (V.) braziliensis* en otros países (noroeste de Argentina y sureste de Brasil) ^44^^,^^45^, con lo que se cumplen los criterios citados por Killick-Kendrick (1988).

En cuanto al área fronteriza con Bolivia, entre 2008 y 2011, se informaron tres casos de leishmaniasis visceral en humanos y en perros infectados en una zona de vegetación xerófila, selvática o rural dispersa a muy pocos kilómetros de Bolivia, sin registro de *Lu. longipalpis*, situación muy similar a la descrita en las primeras décadas del siglo XX en la zona ^11^^,^^46^^,^^47^. Sin embargo, en Tartagal, a 55 km de la frontera, se registró *Lu. longipalpis* de distribución urbana ^48^^,^^49^, con un patrón parecido al observado en el proceso de dispersión del nordeste de Brasil a Argentina entre 1980 y 2004 ^50^. En ese sentido, es interesante que, según sus marcadores genéticos mitocondriales y la diversidad haplotípica, los ejemplares de *Lu. Longipalpis* capturados en Tartagal presentaban características de una población ancestral estable diferente a las de reciente introducción con agrupación propia, quizá perteneciente al dominio amazónico compartido con Bolivia, pero también características de la agrupación Argentina-Brasil en expansión ^50^.

En cuanto a la leishmaniasis visceral, en los cinco (0,7 %) casos positivos según la prueba ELISA rk-39, no se pudo hacer la revisión clínica ni una nueva toma de muestras por el rechazo de los familiares y la muerte de uno de los niños con sintomatología sospechosa ^22^. La coexistencia de infecciones dobles por especies de *Leishmania* y *T. cruzi* debe considerarse al usar métodos de diagnóstico ^43^.

Los entornos urbano-rurales estudiados están rodeados de remanentes de bosque y ofrecen fuentes de alimentos para los vectores (crianza de pollos, cerdos, perros y otros), lo que se añade a la importante actividad agrícola, los bajos índices de desarrollo humano y la ausencia de servicios básicos, todos potenciales factores coadyuvantes para la presencia de enfermedades tropicales.

La información recolectada indica que, aparentemente, el brote inicial de *L.* (*V.*) *braziliensis* en el municipio de Bermejo se está extendiendo en dirección noreste y actualmente abarca otros cuatro municipios (Padcaya, Caraparí, Entre Ríos y Yacuiba), lo que sugiere que *Ny. neivai* se está adaptando al entorno peridoméstico.

Para mitigar el riesgo es necesario aumentar la capacidad de enfrentar la endemia demostrada en el SEDES de Tarija: diagnóstico de laboratorio (no sólo básico), reserva estratégica de medicamentos, vigilancia activa de casos, muestreos entomológicos regulares y, principalmente, sensibilización de la población (trabajadores, empresarios y autoridades), con el fin de plantear estrategias participativas de control y prevención (ropa protectora y hábitos), de diagnóstico temprano, tratamiento oportuno de los casos, desarrollo de modelos predictivos, sistemas de alerta temprana y control de vectores estacionales (con estudios de evaluación antes y después del rociado dentro del domicilio y de la fumigación en el peridomicilio para determinar su efectividad), control de potenciales reservorios (roedores) y manejo de animales domésticos y poblaciones caninas, incluida la tenencia responsable, la capacidad de carga de la localidad y los animales ambulantes.

Las similitudes ecoepidemiológicas y de factores desencadenantes como el clima y la modificación ambiental en la zona de frontera, imponen la necesidad de hacer estudios conjuntos, y coordinar la vigilancia y el manejo de casos entre Bolivia (Tarija-Chuquisaca) y Argentina (Salta-Jujuy). La detección de los grupos y las actividades de riesgo, incluidos los migrantes y la exposición laboral, así como el seguimiento conjunto en ambos países, permitirán mejorar el diagnóstico y el tratamiento, y generar recomendaciones de prevención específicas, no solamente a nivel individual, sino de responsabilidad pública y privada relativas a la regulación, la evaluación del riesgo y la mitigación, la modificación ambiental y el riesgo del efecto de borde (asentamientos, deforestación, cultivos), así como la prevención ante eventos climáticos extraordinarios.
